# Mental health and the COVID-19 pandemic: a study of medical residency training over the years

**DOI:** 10.6061/clinics/2021/e2907

**Published:** 2021-06-23

**Authors:** Vitor S. Mendonça, Amanda Steil, Aécio F.T. Góis

**Affiliations:** I Faculdade de Medicina, FMUSP, Universidade de Sao Paulo, Sao Paulo, SP, BR; II Escola Paulista de Medicina/Universidade Federal de Sao Paulo (EPM/UNIFESP), Sao Paulo, SP, BR

**Keywords:** Medical Residency, Mental Health, COVID-19

## Abstract

**OBJECTIVES:**

This study aims to assess the symptoms of burnout, depression, and anxiety in Brazilian medical residents during the COVID-19 pandemic and to compare residents’ beliefs and clinical practices related to COVID-19 patients among all six years of medical residency training in Brazil.

**METHODS:**

A quantitative study was conducted in April 2020 with a convenience sample of medical resident volunteers from an anonymous online survey. This investigation collected sociodemographic information and used the Oldenburg Burnout Inventory (OLBI) to measure burnout, the Patient Health Questionnaire (PHQ-9) to measure depression, and the General Anxiety Disorders (GAD-7) to measure generalized anxiety disorder. This study also developed a COVID-19 Impact Questionnaire (CIQ-19) to assess the residents’ beliefs and clinical practices related to COVID-19 patients.

**RESULTS:**

Our sample comprised 3071 respondents. Depressive symptoms were the most common among second-year residents (70.5%), followed by anxiety symptoms (56.0%) and burnout (55.2%) among fourth-year residents. We also observed burnout symptoms (55.1%) among second-year residents.

**CONCLUSION:**

The COVID-19 pandemic increased the risk of mental illnesses in some years of residency. Our study could not conclude the reasons why the incidence varies among levels of physician training. Final year medical residents have avoided seeing COVID-19 patients.

## INTRODUCTION

Brazil instituted medical residency in 1977 as the most appropriate form of training for specialist physicians. The country has numerically expanded the training capacity of these physicians in recent years. In 2019, 53,776 physicians studied medical residency in 4,862 programs offered by 809 accredited institutions in Brazil (1).

In Brazil, the duration of medical specialties varies according to the chosen medical field. Therefore, there are physicians in training in the country who spend up to six years in medical residency (1).

COVID-19 was identified in China with a high infectivity and transmissibility rate with a reproductive number greater than one. The world started a very atypical pandemic process as of December 2019 (2-3). Physicians of various specialties were called up to work in the fight against COVID-19 and were reallocated to emergency departments, intensive-care units, and COVID-19 wards to supply the need for medical personnel (3-7).

The psychological effects of the pandemic on healthcare workers must be discussed in medical residency training. The need for a new perspective on mental health care came to light during the last decade, motivated by the high incidence of mental illness and suicides in the medical community (8-10). Risk factors include younger age and less experience, two aspects that may be related to medical residents, a group that needs to achieve the required knowledge and ethical maturity to deal with the difficulties and feelings inherent in this period (3,11).

This study aims to assess the symptoms of burnout, depression, and anxiety in Brazilian medical residents during the COVID-19 pandemic and compare residents’ beliefs and clinical practices related to COVID-19 patients among all six years of medical residency training in Brazil. This study grouped residents according to the year of medical residency training, in an attempt to elucidate the divergences and similarities of each year of training. This is somewhat unprecedented since most studies with medical residents are developed based on the specialty of training. The research seeks to identify specificities in each year of medical residency during the COVID-19 pandemic, regardless of the residency program.

## METHODS

### Study design

A quantitative survey was conducted according to Strengthening the Reporting of Observational Studies in Epidemiology (STROBE) parameters to assess the psychological impact of the COVID-19 pandemic on medical residents from Brazil as they are the ones on the frontline of patient care. This study used a convenience sample from an anonymous online survey advertised on social media and distributed throughout the country by e-mails from residency committees of the universities’ hospitals and national residency and medical associations. Given that a convenience sample was used, no calculation of sample size was performed. Advertisement of the research was performed using good practices guidelines (12). There was no compensation or incentives of any kind for the volunteers.

### Data collection, study measures, and analysis

On February 25, 2020, the Brazilian Ministry of Health confirmed the first case of COVID-19 in the country. The survey was available during April 2020 all over the country. This investigation collected sociodemographic information and used the Oldenburg Burnout Inventory (OLBI) to measure burnout, the Patient Health Questionnaire (PHQ-9) to measure depression, and the General Anxiety Disorders (GAD-7) to measure generalized anxiety disorder (13-15). All three scales were previously adapted and validated for use in the Brazilian context and population. This study also developed a COVID-19 Impact Questionnaire (CIQ-19) to assess the residents’ beliefs and clinical practices related to COVID-19 patients, their behaviors concerning disease prevention, and their mental health care. All fields were marked as mandatory, so a participant could move forward only after answering all questions. Therefore, all included participants completed the entire questionnaire, so no data were missing. The protocol was reviewed and approved by the Universidade Federal de São Paulo, Brazil (UNIFESP) Research Ethics Committee (Protocol#3,943,348 on March 20, 2020).

First, this investigation conducted exploratory analyses using basic contingency tables with Anova, Mann-Whitney U test, and Fisher’s test. The residents’ sociodemographic variables, characteristics of the residence program, clinical practice and beliefs of COVID-19, and mental health care were described. Burnout was defined as positive if the total score on OLBI was 21; anxiety was defined as positive if the total score on GAD-7 was ten or greater. PHQ-9 scores were categorized as: (1) no depression or mild depression for scores less or equal to nine; (2) moderate for scores between 10 and 14; and (3) severe depression for scores higher or equal to 15.

All frequencies analyses were controlled by year of medical residency (from first to sixth year - R1 to R6), gender, specialty (clinical specialties, surgical specialties, and diagnostic and therapeutic support (i.e., pathology, radiotherapy, and nuclear medicine)), region of Brazil, nature of the hospital (public or private) and contact with COVID-19 patient. Analyses were performed using SPSS Statistics for Windows, Version 22.0 (released 2013, IBM Corp, Armonk, NY, USA) with a level of statistical significance as 0.05. The results are presented as proportions and the distribution of the scores in the categories of each scale (frequencies).

## RESULTS

Our sample comprised 3071 residents from all over the country. Approximately, the response rate represents 10% of medical residents in Brazil. Most were women (75.3%), white (77.7%), residents of clinical specialties (65.4%) held in programs provided by a public hospital (85.6%) in southeast Brazil (63.8%), and had contact with COVID-19 patients (62.7%). The mean age of the sample was 28.0 years old (SD:3.2), and all 26 Brazilian states and the federal district were represented (Table 1).

Our findings showed statistical significance when analyzing mental health scales, COVID-19 aspects of clinical practice, and mental health care across all six years of medical residency training.

Regarding sample characteristics by year of training, we had the majority of the residents in the first year R1 (n=1121). Depressive symptoms were the most common among R2 medical residents (70.5%), followed by anxiety symptoms (56.0%) and burnout (55.2%) among R4 residents. We also observed burnout symptoms (55.1%) among R2 medical residents (Table 2).

In terms of clinical practice, R3 residents were more likely to feel that the hospital was prepared to treat patients with this disease (81.8%). Avoiding seeing patients with confirmed or suspected cases of COVID-19 was more common among R5 and R6 medical residents (77.1% and 78.5%, respectively). The following aspects were related to the lack of supervisor support for the treatment of COVID-19 patients. 86.7% of R5 felt that the COVID-19 situation would impact their training, and R1 and R3 residents worked in wings with high risk of contamination (74.3% and 75.3%, respectively) (Table 3).

The provision of personal protection equipment (PPE) by the hospital was not efficacious for the residents from the 2nd and 3rd year of training (26.5% and 36.1%, respectively), as it was for R5 medical residents (71.0%). First-year residents felt significantly worried about getting COVID-19 and transmitting it to significant others (98.4%), and the same group also had more personal relationships impaired since the pandemic (84.4%) (Table 3).

Finally, our sample revealed some responses associated with mental health care among all groups. These physicians preferred to talk with family or friends (37.8%) and discuss with their support team (20.2%) when they needed mental health care. R5 and R6 residents had high rates of physical activity as a form of mental health care (39.3% and 42.8%, respectively). Individuals in the R6 group did nothing about their mental health care (33.3%), and only 7.7% among all the years referred psychotherapy regarding their personal or professional issues (Table 4).

## DISCUSSION

This study analyzed the symptoms of burnout, depression, and anxiety in Brazilian medical residents during the COVID-19 pandemic among residents according to the year of medical residency training. Our results indicate that depressive symptoms were most common among R2 residents, followed by anxiety symptoms and burnout among R4 residents. R3 residents were more likely to feel that the hospital was prepared to treat patients with this disease. Residents in the first year of training felt significantly worried about getting COVID-19 and transmitting it to significant others. As a form of mental health care, residents from all years of training preferred to talk with family or friends, while residents in the sixth year of training had higher rates of physical activity when compared to the other groups.

Concerning the universe of medical residents in Brazil, a previous study published in 2019 observed that physicians were mostly women (55%), reflecting the trend of the feminization of medicine in Brazil. We could also see in the findings of this study that 75.3% of respondents were women. In almost all years of this study, the number of women was higher than that of men, except for the sixth year of residency. The previously cited study also reports that physicians in training are still unevenly distributed across the Brazilian territory, as are institutions and programs offering medical residency positions. The Southeast region has 57.3% of resident physicians, representing more than half of the entire country. It is also concentrated with more than half of the authorized programs in the country. Our findings corroborate the data from the previous study, considering that we also noted an irregular distribution of medical residents across Brazil, with a strong tendency for concentration to the southeast region (1).

In almost all years of medical training, most residents pursue clinical specialties, except for sixth-year residents, the vast majority of whom are surgical specialists. A review of Brazilian medical residents indicated 16,190 physicians in R1, 15,453 in R2, and 119 in R6. A little more than 20,000 physicians were in clinical specialties in Brazil in 2019 (1). Most years had contact with patients with COVID-19. Once again, the exception was R6, as it is a year of the specialty more focused on complex surgeries.

The mental health of medical residents is already a topic that has worried medical educators worldwide even before the pandemic scenario emerged. In 2014, the prevalence rates for anxiety, depression, and burnout among Brazilian residents were 41.3%, 21.6%, and 58.4%, respectively. Therefore, symptoms of anxiety increased two-fold, and symptoms of depression exhibited an approximately three-fold increase in the COVID-19 pandemic scenario among overall participants. However, the prevalence of burnout identified in R2 and R4 physicians training is similar to that of a non-pandemic scenario. Then, we cannot say whether this situation is an intrinsic case only in R2 and R4 groups or an effect of the pandemic. R4 group residents also showed a higher rate of anxiety when compared overall (16).

These findings are similar to those of a survey of 1,257 health care workers in contact with COVID-19 patients in China, which reported high rates of depression (50.4%), anxiety (44.6%), and distress (71.5%), using the same instruments as those used in this study. We also observed a high rate of depression among R2 physicians training in Brazil (17). Depressive symptoms and the prevalence of burnout among the R2 group reinforce the fact that the novice and younger residents seem to suffer the most from the COVID-19 pandemic scenario.

Our study also showed that the way Brazilian medical residents cope with mental disorders is more focused on talking with family and friends in general, and we observed that 37.8% of physicians practice this as a form of mental health care. Between the R5 and R6 groups, there is a representative rate of physicians who practice physical activity with a focus on mental health care. We noted a greater presence of men and physicians of surgical specialties in the R6 group, which could explain the high incidence of this practice. However, in the same R6 group, there is a high rate of participants who do not practice mental health care, and there are few residents who engage in any form of psychotherapy activity.

Given these findings concerning the development of mental disorder symptoms, we propose the following interventions to prevent mental disease in this group. Studies highlight the importance of giving attention to physicians who avoid contact with COVID-19 patients. Medical institutions need to understand how stressful the current situation is to implement care measures. This includes clear communication with staff, provision of protective measures and PPEs, and sensitive administration of work shifts (1,18-19).

To address these issues, the medical institution can also offer mental health and psychosocial support, and psychological/psychiatric treatment to its employees. This situation is based on Mental Health and Psychosocial Support (MHPSS) in emergency settings to promote a “local or outside support that aims to protect psychosocial well-being and/or prevent or treat mental health conditions” (20).

In general, these residents did not feel prepared to treat COVID-19 patients, even though we observed a slight confidence from R3. Perhaps this is justified by the fact that they work in an environment with a high risk of contamination and that institutions had a lack of PPE, leading to increased fear of getting COVID-19, transmitting it to someone significant, or impacting social relations. Thus, physicians need to gain more confidence as they see more patients with the disease, have adequate support from supervisors to treat this disease, and PPE is made available in the hospitals. This notion explains that residents would become better professionals after the pandemic, as they treat more and more patients with this disease over time (21).

On the other hand, there is a high proportion of R5 residents who missed supervisor's support for the treatment of COVID-19. Perhaps this is why these same residents avoided seeing patients with confirmed or suspected cases of the disease. However, the same logic does not apply to the R6 residents who also had high rates of avoidance of these patients, but did not miss the support of supervisors. Previous studies analyzed the psychological effects of emerging virus outbreaks on healthcare workers and found that staff in high-risk areas exhibited increased levels of acute or posttraumatic stress and psychological distress. The supervisor is an important person to discuss challenging ethical situations with, sharing the burden of tough decisions and helping the resident to gain experience and confidence (11,22).

This study design is adequate to investigate associations and provide wide-ranging data for discussion but does not allow for inferences of causality. In a way, some differences found between years of residency may seem more like the structure of medical residency programs in Brazil, such as younger residents and beginners. Perhaps, if the final sample was not small, we could accurately associate the relationship between residents' mental suffering and the COVID-19 pandemic scenario. In addition, since our sample was restricted to Brazil, further studies should investigate whether the trend cited above is replicable in other countries among different years of medical residency. Therefore, we must be cautious with generalizations of these results to distinct populations. Another limitation is that the COVID-19 Impact Questionnaire is not validated. It was created by a multidisciplinary team with professionals working on the COVID-19 scene and based on previous studies in other pandemics.

## CONCLUSION

Considering the still-evolving situation of COVID-19, every year of medical residency has the same experience with the disease and these residents may be at risk for mental illnesses, such as depression, anxiety, and burnout. However, the novice and younger residents seem to suffer the most from this impact, perhaps due to the structure of the medical residency, as it was these groups that were most on the front line. More specialized residents (R5 and R6 groups) avoided seeing patients with COVID-19, hence, it is important that supervisors continue to provide support regarding the care of patients with COVID-19, even for physicians in advanced residency training.

This study also highlights the general need for better access to mental health professionals for resident physicians who worked during the COVID-19 pandemic. It is proposed that health institutions give medical supervisors a closer and more unique look at physicians in training.

## AUTHOR CONTRIBUTIONS

Mendonça VS, Steil A and Góis AFT conceived and designed the study. Mendonça VS and Steil A led the development of the credibility instrument. All authors participated in the data collection. Mendonça VS conducted the data analysis and drafted the initial version of the manuscript. All authors contributed to the interpretation of the data and critically revised the manuscript. All authors had full access to tables in the study and take responsibility for the integrity of the data and the accuracy of the data analysis.

## Figures and Tables

**Table 1:** Descriptive statistics of medical residents from Brazil, 2020.

		Years of medical residency														
		R1 n=1121		R2 n=976		R3 n=600		R4 n=277		R5 n=83		R6 n=14		Total n=3071		
Descriptive		n	%	n	%	n	%	n	%	n	%	n	%	n	%	*p*-value
Gender																>0.05
	Male	283	25.2	219	22.4	156	26.0	61	22.0	33	39.7	8	57.1	760	24.7	
	Female	838	74.7	757	77.5	444	74.0	216	78.0	50	60.2	6	42.8	2311	75.3	
Ethnicity																>0.05
	White	883	78.7	761	78.0	457	76.1	203	73.2	70	84.3	12	85.7	2386	77.7	
	Non-white	238	21.2	215	22.0	143	23.8	74	26.7	13	15.6	2	14.2	665	22.3	
Region of Brazil																>0.05
	Southeast	705	62.8	603	61.7	378	63.0	194	70.0	68	81.9	13	92.8	1961	63.8	
	South	168	15.0	160	16.3	96	16.0	28	10.1	10	12.0	-	-	462	15.0	
	Northeast	142	12.6	103	10.5	63	10.5	33	11.9	5	6.0	1	7.1	347	12.0	
	Midwest	85	7.5	75	7.6	46	7.6	17	6.1	-	-	-	-	223	7.2	
	North	21	1.8	35	3.5	17	2.8	5	1.8	-	-	-	-	78	2.5	
Hospital nature																**<0.05**
	Public	952	84.9	827	84.7	527	87.8	247	89.1	64	77.1	11	78.5	2628	85.6	
	Private	169	15.0	149	15.2	73	12.1	30	10.8	19	22.8	3	21.4	443	14.4	
Specialty^a^																**<0.05**
	Clinical specialties	755	67.3	649	66.4	331	55.1	221	79.7	48	57.8	5	35.7	2009	65.4	
	Surgical specialties	343	30.5	293	30.0	219	36.5	51	18.4	33	39.7	8	57.1	947	30.8	
	Diagnostic and therapeutic support	41	3.6	34	3.4	38	6.3	1	0.3	1	1.2	-	-	115	3.7	
Has contact with COVID-19 patients																**<0.05**
	Yes	719	64.1	646	66.1	358	59.6	152	54.8	45	54.2	6	42.8	1926	62.7	
	No	402	35.8	330	33.8	242	40.3	125	45.1	38	45.7	8	57.1	1145	37.2	

a as described in the Methods section.

**Table 2:** Mental health scales scores among medical residents from Brazil, 2020.

		Years of medical residency														
		R1 n=1121		R2 n=976		R3 n=600		R4 n=277		R5 n=83		R6 n=14		Total n=3071		
Mental Health		n	%	n	%	n	%	n	%	n	%	n	%	n	%	*p* value
Depression^a^																**<0.05**
	absent or mild	382	34.0	287	29.4	198	33.0	89	32.1	29	34.9	7	50.0	992	32.3	
	moderate	326	29.0	280	**28.6**	142	23.6	67	24.1	22	26.5	4	28.5	841	27.4	
	severe	413	36.8	409	**41.9**	260	43.3	121	43.6	32	38.5	3	21.4	1238	40.3	
Anxiety^b^																**<0.05**
	absent or mild	554	49.4	448	45.9	271	45.1	122	44.0	44	53.0	9	64.2	1448	47.2	
	moderate or severe	567	50.5	528	54.0	329	54.8	155	**56.0**	39	47.0	5	35.7	1623	52.8	
Burnout^c^																**<0.05**
	absent or mild	658	58.6	438	44.8	305	50.8	124	44.7	43	51.8	11	78.5	1579	51.4	
	moderate or severe	463	41.3	538	**55.1**	295	49.1	153	**55.2**	40	48.1	3	21.4	1492	48.6	

a according to the Patient Health Questionnaire - 9;

b according to the General Anxiety Disorder - 7;

c according to the Oldenburg Burnout Inventory.

**Table 3:** Frequency of COVID-19 clinical practice and beliefs among medical residents from Brazil, 2020.

		Years of medical residency														
		R1 n=1121		R2 n=976		R3 n=600		R4 n=277		R5 n=83		R6 n=14		Total n=3071		
COVID-19 clinical practice and beliefs		n	%	n	%	n	%	n	%	n	%	n	%	n	%	*p* value
	Feel prepared to treat patients of COVID-19	319	28.4	336	34.4	491	**81.8**	113	40.7	51	61.4	9	64.2	1319	43.0	**<0.05**
	Avoiding seeing patients with confirmed or suspected cases of COVID-19	351	31.3	223	22.8	298	49.6	94	33.9	64	**77.1**	11	**78.5**	1041	33.9	**<0.05**
	Supervisors do not provide the necessary support for the treatment of COVID-19 patients	429	38.2	314	32.1	287	47.8	107	38.6	72	**86.7**	4	28.5	1213	39.4	**<0.05**
	Work in a wing with high risk of contamination with COVID-19	834	**74.3**	694	71.1	452	**75.3**	176	63.5	47	56.6	2	14.2	2205	71.8	**<0.05**
	Hospital provides personal protection equipment	477	42.5	259	26.5	217	36.1	155	55.9	59	**71.0**	7	50.0	1174	38.2	>0.05
	Afraid of getting COVID-19 and transmitting it to my significant ones	1104	**98.4**	931	95.3	538	89.6	198	71.4	57	68.6	12	85.7	2840	92.5	>0.05
	Personal relationships impaired since the pandemic	947	**84.4**	601	65.1	505	****84.1****	192	69.3	55	66.2	3	21.4	2303	75.0	**<0.05**

**Table 4:** Mental health care score of medical residents from Brazil, 2020.

		Years of medical residency														
		R1 n=1121		R2 n=976		R3 n=600		R4 n=277		R5 n=83		R6 n=14		Total n=3071		
		n	%	n	%	n	%	n	%	n	%	n	%	n	%	*p* value
Mental Health Care^a^																**<0.05**
	talk with friends/family	937	**44.1**	678	39.0	401	33.5	152	27.8	24	13.1	3	14.2	2195	**37.8**	
	support team	607	28.5	328	18.9	104	8.7	112	20.5	23	12.5	-	-	1174	20.2	
	physical activity	140	**6.5**	276	15.9	221	18.5	103	18.8	72	**39.3**	9	**42.8**	821	14.1	
	nothing	293	13.7	217	12.5	144	12.0	41	7.5	25	13.6	7	**33.3**	727	**12.5**	
	psychotherapy	95	4.4	142	8.1	116	9.7	71	13.0	23	12.5	-	-	447	**7.7**	
	others	52	2.4	94	5.4	208	17.4	66	12.2	16	8.7	2	9.5	438	7.5	
Total responses		2124	36.6	1735	29.9	1194	20.6	545	9.4	183	3.1	21	0.3	5802	100.0	

a multiple responses.
